# Follow-up short and long-term mortalities of tracheostomized critically ill patients in an Italian multi-center observational study

**DOI:** 10.1038/s41598-024-52785-y

**Published:** 2024-01-28

**Authors:** Maria Vargas, Denise Battaglini, Massimo Antonelli, Ruggero Corso, Giulio Frova, Guido Merli, Flavia Petrini, Marco V. Ranieri, Massimiliano Sorbello, Ida Di Giacinto, Pierpaolo Terragni, Iole Brunetti, Giuseppe Servillo, Paolo Pelosi

**Affiliations:** 1 Department of Neurosciences, Reproductive and Odontostomatological Sciences, University of Naples “Federico II”, Via Pansini, 80100 Naples, Italy; 2 IRCCS Ospedale Policlinico San Martino, Genova, Italy; 3 Dip Scienze dell’Emergenza, Anestesiologiche e della Rianimazione, Fondazione Policlinico Universitario A. Gemelli IRCCS, Rome, Italy; 4 Istituto di Anestesiologia e Rianimazione, Università Cattolica del Sacro Cuore, Rome, Italy; 5 Dipartimento delle Terapie Intensive, Anestesiologia e Terapia del Dolore, Ospedale “Guglielmo da Saliceto”-Piacenza, Piacenza, Italy; 6 Università degli Studi di Milano and Dipartimento Anestesia e Rianimazione Spedai Civili si Brescia, Brescia, Italy; 7 U.O.C. Anestesia e Rianimazione Ospedale Maggiore di Crema, Asst Crema, Italy; 8 Coordinamento Strutturale Medicina Perioperatoria, Terapia Dolore, Emergenze Intraospedaliere, Terapia Intensiva – ASL2 Abruzzo, Università di Chieti-Pescara, Pescara, Italy; 9 Alma Mater Studiorum-Università di Bologna, IRCCS Policlinico di Sant’Orsola, Anesthesia and Intensive Care Medicine, Bologna, Italy; 10 UOC Anestesia e Rianimazione, Giovanni Paolo II Hospital, Ragusa, Italy; 11 Anesthesia and Intensive Care, Mazzoni Hospital, Ascoli Piceno, Italy; 12 Department of Medicine, Surgery and Pharmacy, University of Sassari, Sassari, Italy; 13 Department of Surgical Sciences and Integrated Diagnostics, University of Genoa, Genoa, Italy

**Keywords:** Quality of life, Outcomes research

## Abstract

The effects of tracheostomy on outcome as well as on intra or post-operative complications is yet to be defined. Admission of patients with tracheostomy to rehabilitation facility is at higher risk of suboptimal care and increased mortality. The aim of the study was to investigate ICU mortality, clinical outcome and quality of life up to 12 months after ICU discharge in tracheostomized critically ill patients. This is a prospective, multi-center, cohort study endorsed by Italian Society of Anesthesia, Analgesia, Reanimation, and Intensive Care (SIAARTI Prot. n° 643/13) registered in Clinicaltrial.gov (NCT01899352). Patients admitted to intensive care unit (ICU) and requiring elective tracheostomy according to physician in charge decision were included in the study. The primary outcome was ICU mortality. Secondary outcomes included risk factors for ICU mortality, prevalence of mortality at follow-up, rate of discharge from the hospital and rehabilitation, quality of life, performance status, and management of tracheostomy cannula at 3-, 6, 12-months from the day of tracheostomy. 694 critically ill patients who were tracheostomized in the ICU were included. ICU mortality was 15.8%. Age, SOFA score at the day of the tracheostomy, and days of endotracheal intubation before tracheostomy were risk factors for ICU mortality. The regression tree analysis showed that SOFA score at the day of tracheostomy and age had a preeminent role for the choice to perform the tracheostomy. Of the 694 ICU patients with tracheostomy, 469 completed the 12-months follow-up. Mortality was 33.51% at 3-months, 45.30% at 6-months, and 55.86% at 12-months. Patients with tracheostomy were less likely discharged at home but at hospital facilities or rehabilitative structures; and quality of life of patients with tracheostomy was severely compromised at 3–6 and 12 months when compared with patients without tracheostomy. In patients admitted to ICU, tracheostomy is associated with high mortality, difficult rehabilitation, and decreased quality of life. The choice to perform a tracheostomy should be carefully weighed on family burden and health-related quality of life.

**Clinical trial registration**: Clinicaltrial.gov (NCT01899352).

## Introduction

Tracheostomy is a common procedure in intensive care unit (ICU) to facilitate the weaning from respiratory support in patients who require long-term invasive mechanical ventilation^1,2^. Few clinical guidelines have been developed to suggest the best practice for this invasive and risky procedure, however surveys performed on this topic in different European countries have shown the presence of a shared clinical practice^3^.

Further, information on the effect of tracheostomy on clinically relevant outcomes and quality of life after ICU discharge is scanty^4^. Patients who require prolonged ventilatory support can be at higher risk of unfavorable outcome. The effects of tracheostomy on outcome as well as on intra or post-operative complications may depend on the severity and type of disease^5–8^. Patients with tracheostomy are often discharged from the ICU to a general ward or rehabilitation facility with higher risk of suboptimal care and increased mortality^9,10^. Individualized plans for tracheostomized patients as well as post-discharge follow-up focused on quality of life may improve patient outcome and safety^11^.The aim of the study was to investigate ICU mortality, clinical outcome and quality of life up to 12 months after ICU discharge in tracheostomized critically ill patients.

## Methods

This is a prospective, multi-center, cohort study endorsed by Italian Society of Anesthesia, Analgesia, Reanimation, and Intensive Care (SIAARTI Prot. n° 643/13) registered in Clinicaltrial.gov (NCT01899352—15 July 2013). The institutional ethical committee (EC) of the coordinating center approved the study protocol (university of Genoa 53/12) and all the methods and procedures of this study were performed in accordance with the relevant guidelines and regulations. After the approval by the EC of the coordinator center, each local institutional ethic committee approved the study protocol. The STROBE Statement—checklist was applied for this study. The list of participating centers is available in Table S1—supplementary material. The recruitment of patients was performed from 2014 and 2015 while the follow-up period lasted until the 2016. Data from each center were received in the late 2018. Then, since the data collection was performed with manual system, we had to check carefully each database before starting the statistical analysis. Patients aged more than 18 years old, admitted to the ICU and requiring elective tracheostomy were consecutively included in the study after obtaining the informed consent according to local regulations. Patients with severe neck infections, recent major neck surgery or cervical spine surgery were excluded. During the recruitment period, data about patient characteristics, comorbidities, reason of ICU admission, airway evaluation, type and procedures of tracheostomies, intraprocedural, early and late complications, discharge and mortality were recorded. Follow-up was performed at 3-months, at 6-months and 1-year from the day of tracheostomy. Patients were contacted by phone for a medical interview. The medical interview included the evaluation of questionnaire of Euro Quality of Life questionnaire (EQ-5D-5L), performance status scale for head and neck surgery, management of tracheostomy cannula and mortality. The primary outcome of the study was the ICU mortality. The secondary outcomes were the evaluation of: (1) risk factors for ICU mortality; (2) mortality at 3-, 6-, 12-months and follow-up; (3) the quality of life at 3-, 6-, 12- months from the day of tracheostomy.

### Statistical analysis

Data are reported as mean and standard deviation (± SD), median (interquartile ranges, IQR), or proportion as appropriate. A two-way ANOVA for continuous variables or chi-square test for categorical variables was used for comparisons between groups. For the analysis, we divided patients in survivors and non-survivors in ICU. A logistic-regression model was used to identify the predictors of ICU mortality. Factors with p < 0.1 in the univariate analysis were entered into the multivariate models. Thereafter, multiple logistic regressions were carried out using backward stepwise variable elimination. A classification of regression tree (CRT) analysis was also made to characterize risk groups for ICU mortality. The CRT approach was employed to generate a classification tree prediction algorithm. The CRT analysis determines optimal cutoffs for investigated variables and results in a classification tree. The resulting classification tree was then used as a prediction algorithm and applied to the test dataset. For the follow-up analysis, we divided patients in still tracheostomized at 3 months (Tracheostomy group) and non-tracheostomized (no Tracheostomy group). Mortality during follow-up period was calculated as prevalence, meaning the ratio between the progressive sum of patients who died divided by the total number of patients. Same computation was made for discharge data. Student’s t-test or Mann–Whitney *U*-tests and chi-square test were used to compare continuous and categorical variables, respectively. Median differences (MD) between time points were computed using the Hodges–Lehmann estimator with their 95% confidence interval (CI). Statistical significance (p) was set at 0.05. Statistical analysis was obtained with SPSS (version 20.0 and 23.0, IBM®, USA), graphical presentation of data was obtained with GraphPad Prism (version 8.0).

### Ethics approval

The institutional ethical committee of the coordinating center approved the study protocol (university of Genoa 53/12).

### Consent to participate

Informed consent for this study was obtained from all individual participants or from the parents.

## Results

### Patient characteristics, ICU mortality, tracheostomy procedures and complications

Six hundred and ninety-four critically ill patients who were tracheostomized in ICU were consecutively included in this study. The main characteristics of the included patients are reported in Table 1. Patients were recruited by 15 Italian ICUs participating in this study. One hundred and ten (15.8%) tracheostomized patients died in ICU while 497 (71.6%) patients survived, 87 patients were lost at follow-up. Survivors compared with non survivors had a lower age, lower SAPS II, APACHE II and SOFA score at the ICU admission, a lower SOFA score at the day of the tracheostomy and less days of mechanical ventilation (Table 1). Tracheostomized patients admitted in ICU for acute respiratory failure and acute renal failure had higher mortality compared to traumatized patients (Table 1). The tracheostomy techniques and procedural findings, intraprocedural early and late complications of included patients are shown in Tables S2 and S3 supplementary material. Data about patients discharged alive from ICU are shown in Fig. S1—supplementary material. Four hundred and ninety-seven tracheostomized patients (52.5%), were discharged alive from ICU to medical wards (52.5%), 114 patients (22.9%) to surgical wards and 122 patients (24.5%) to physical therapy/rehabilitation. Patients with mechanical ventilation were mainly transferred in medical wards rather than in surgical wards or physical therapy/rehabilitation.

**Table 1:** Characteristics of patients included in the study.

	Survivors (n = 497)	Non-survivors (n = 110)	p
Age (years)	62 ± 17	68 ± 13	0.001
Sex (F/M)	194/303	37/73	0.456
BMI (Kg/m^2^)	25 ± 7	26 ± 4	0.572
SAPS II	49 ± 17	53 ± 16	0.019
APACHE II	20 ± 9	23 ± 7	0.009
SOFA at intensive care unit admission	7 ± 3	9 ± 6	0.000
SOFA at the day of tracheostomy	6 ± 3	7 ± 4	0.000
SOFA at intensive care unit discharge	3 ± 2		
Causes of intensive care unit admission (n/%)			
Acute respiratory failure	256 (53.8%)	77 (70%)	0.002
Hepatic failure	12 (26.6%)	4 (3.6%)	0.521
Renal failure	42 (8.8%)	18 (16.3%)	0.019
Acute cardiovascular failure	75 (15.7%)	26 (23.6%)	0.05
Chronic cardiac failure	32 (6.7%)	11 (10%)	0.238
Coma	115 (24.7%)	19 (19%)	0.129
Sepsis	81 (17%)	24 (21.8%)	0.241
Trauma	82 (17.1%)	10 (9.1%)	0.034
Neurologic disease	138 (29%)	28 (25.4%)	0.452
Timing of tracheostomy (d)	9 ± 6	11 ± 7	0.003
Days of mechanical ventilation (d)	11 ± 8	12 ± 8	0.760
Length of intensive care unit stay (d)	12 ± 2	13 ± 9	0.329

Data are reported as mean ± SD or percentage as appropriate. *d* days, *BMI* body mass index, *SAPS II* simplified acute physiology score II, *SOFA* sequential organ failure assessment score, *APACHE II* Acute Physiologic Assessment and Chronic Health Evaluation II.

### Regression analysis and regression tree for ICU mortality

**Figure 1:**
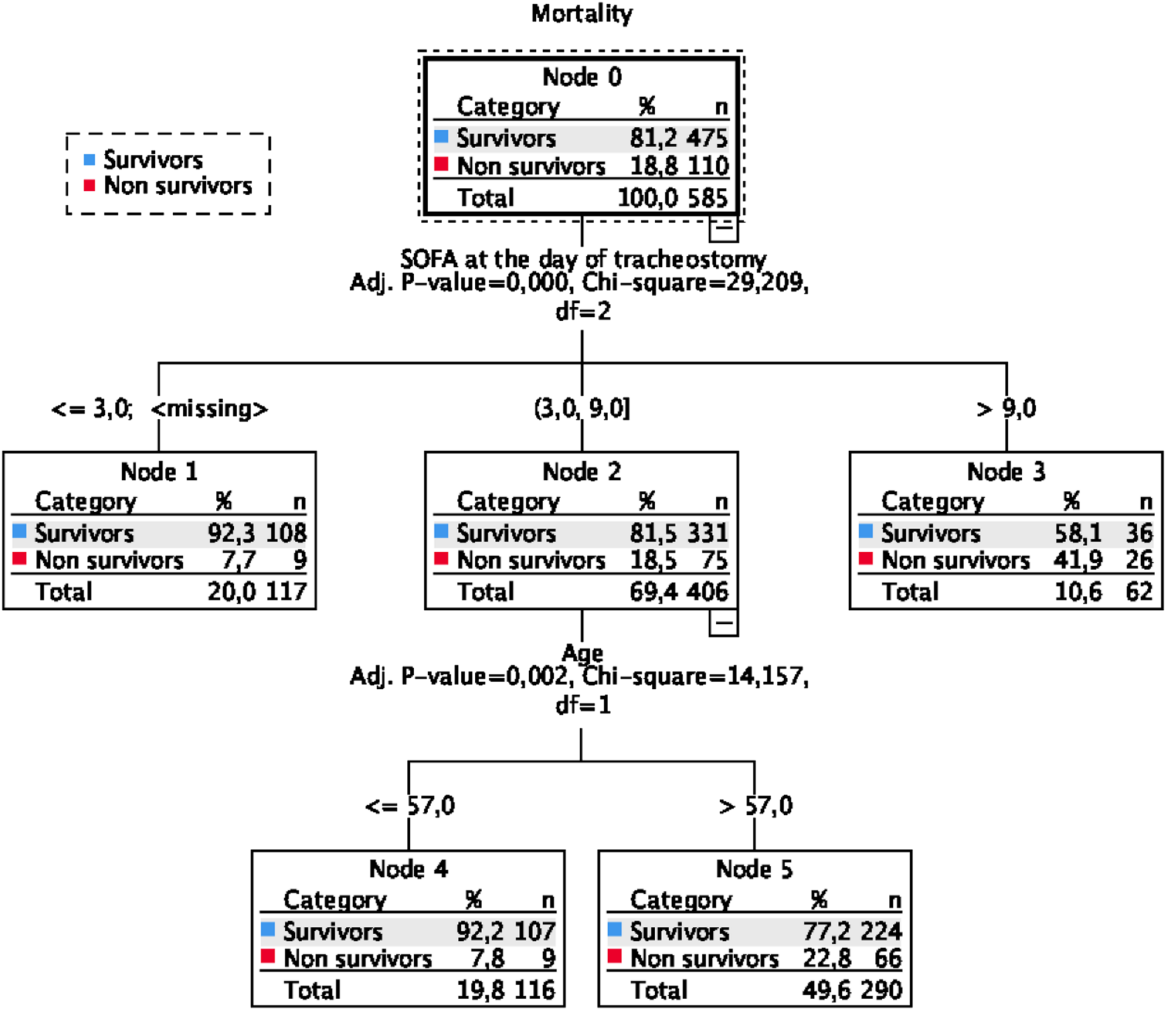
Regression tree analysis depicting the risk groups for ICU mortality. For a SOFA score at the day of tracheostomy ≤ 3 points the risk for ICU mortality was 7.7% (node 1), for a SOFA score at the day of tracheostomy between 3 and 9 an age > 57 years the risk for ICU mortality was 49.6% (node 2 and 5), while SOFA score at the day of tracheostomy > 9 points the risk for ICU mortality was 10.6% (node 3).

In a multivariate analysis, ICU mortality was independently associated with age (OR 1.03; p = 0.019), SOFA score at the day of tracheostomy (OR 1.18; p = 0.000) and days of endotracheal intubation (OR 1.05; p = 0.013) (Table 2). Figure 1 shows the regression tree analysis depicting the risk groups for ICU mortality. For a SOFA score at the day of tracheostomy ≤ 3 points the predicted risk for ICU mortality was 7.7%, for a SOFA score at the day of tracheostomy between 3 and 9 an age > 57 years the predicted risk for ICU mortality was 22.6%, while SOFA score at the day of tracheostomy > 9 points the predicted risk for ICU mortality was 41.9%.

**Table 2:** Factors independently associated with ICU mortality in tracheostomized patients by multiple logistic regression model.

	OR	95% CI	p
Age (years)	1.025	1.004–1.046	0.019
SOFA at the ICU admission	1.061	0.998–1.127	0.059
APACHE II at the ICU admission	0.938	0.943–1.025	0.416
SOFA at the day of tracheostomy	1.177	1.083–1.279	0.000
Timing of tracheostomy	1.046	1.009–1.043	0.013
Acute respiratory failure	1.597	0.920–2.771	0.096
Acute renal failure	1.789	0.887–3.774	0.561
Trauma	0.544	0.256–1.156	0.114

*OR* Odds Ratio, *CI* Confidence interval, *SOFA* sequential organ failure assessment score, *APACHE II* Acute Physiologic Assessment and Chronic Health Evaluation II.

### Follow-up and mortality

A total of 174 patients were lost at follow-up, 91 at 3-months, 31 at 6-months, and 52 at 12-months).

Overall, 185 patients (33.5%) died at 3-months, 236 patients (45.3%) died at 6-months, and 262 patients (55.9%) died at 12-months. Among patients alive with data on tracheostomy, at 3-months follow-up 169 patients (46.2%) still had a tracheostomy cannula and 197 patients (53.8%) did not have a tracheostomy cannula. At 6-months follow-up 63 patients (22.5%) still had a tracheostomy cannula and 217 patients (77.5%) did not. At 12-months follow-up 36 patients (17.5%) still had a tracheostomy cannula and 170 patients (82.5%) were without tracheostomy Baseline characteristics of the patients at 3-, 6-, 12-months from the day of tracheostomy are reported in Table S4—supplementary material. Characteristics of patients with tracheostomy at 3-, 6-, 12- months follow up are reported in Table S5—supplementary material.

### Discharge from hospital and rehabilitation

At 3, 6, and 12 months 123 (57.5%), 172 (64.7%), 173 (84.0%) patients were discharged at home; 93 (26.5%), 29 (10.9%), 5 (2.4%) patients were in a hospital facility, and 146 (41.6%), 66 (24.8%), 25 (12.2%) patients in a rehabilitation ward, respectively.

Tracheostomy group was significantly less likely to be discharged at home, more likely to be admitted to a hospital facility at 3-months and more frequently admitted to a rehabilitative structure at 3–6–12 months than no Tracheostomy group [p < 0.001].

### Quality of life

**Figure 2:**
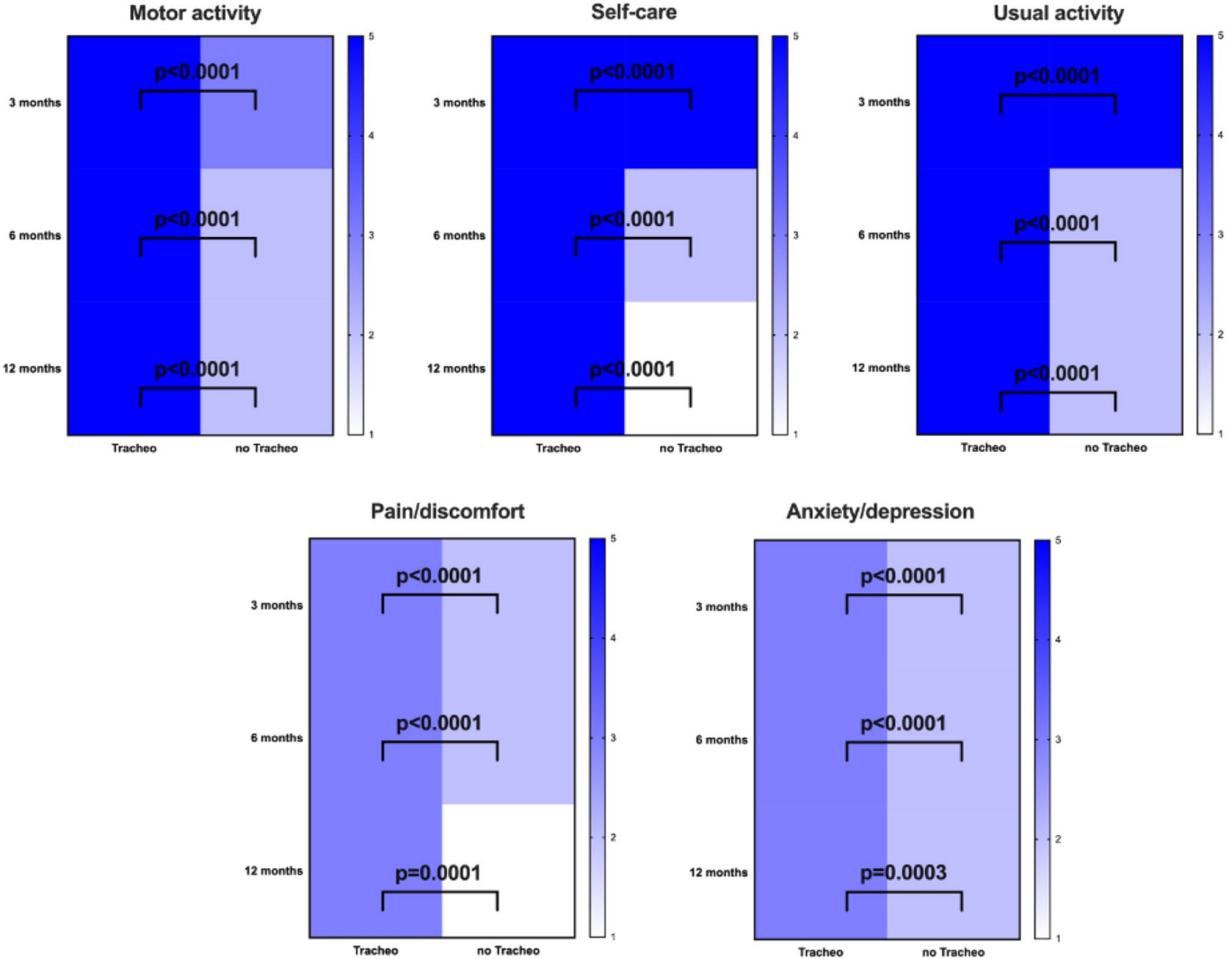
Heatmap of each dimension of the EQ-5D-5L. Median values are compared at 3–6–12 months between Tracheostomy and no tracheostomy (noTracheo) groups. The darker the color, the higher the value (up to 5 points for each domain).

**Figure 3:**
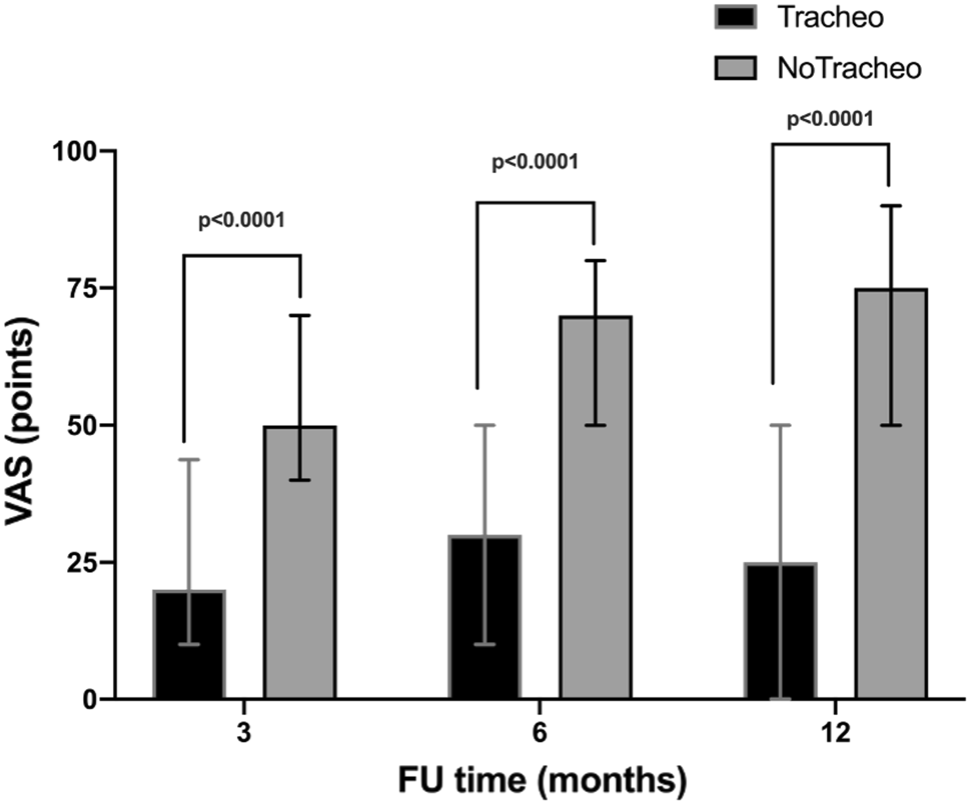
Comparison of median points of VAS between tracheostomy and no tracheostomy (noTracheo) groups at 3-, 6- and 12 months.

In the overall population, the motor activity, self-care, usual activity, pain/discomfort, anxiety/depression and VAS improved over time [3 vs 12 months, p < 0.001]. At 3-, 6-, and 12-months, the median of each dimension of the EQ-5D-5L was improved in patients without Tracheostomy compared to those with Tracheostomy, Fig. 2. The frequency of response for each dimension of the EQ-5D-5L is depicted in Fig. S2—supplementary material. At 3-, 6-, and 12-months, VAS improved in patients without Tracheostomy, Fig. 3.

## Discussion

In critically ill patients admitted to ICU and undergoing elective tracheostomy, we found that: (1) ICU mortality was 15.8% while mortality at 3-months was 33.5%, 45.3% at 6-months, and 55.9% at 12-months; (2) age, SOFA score at the day of the tracheostomy and days of endotracheal intubation were risk factors for ICU mortality; (3) in the regression tree analysis the SOFA score at the day of tracheostomy and age had the main role in predicting the risk for mortality; (4) patients with tracheostomy were less likely discharged at home but at hospital facilities or rehabilitative structures; and (5) quality of life of patients with tracheostomy was severely compromised at 3–6 and 12 months when compared with patients without tracheostomy.

This is the first multicenter observational study evaluating the procedures, complications, and follow-up in a large sample of tracheostomized critically ill patients admitted to ICUs in Italy.

In this study we found an increasing mortality from ICU to 12-months, in line with previous monocentric Italian data that reported a progressively increasing mortality from 47 to 71% over 1 years^4^. Other studies have reported contrasting data on mortalities during the follow-up of critically ill patients who required tracheostomy in ICU. Compared to our results, Cinotti et al. reported a slightly lower mortality at 12-months, 45.2% in patients with tracheostomy and 51.5% in patients without tracheostomy^12^. However, mortalities in tracheostomized patients may change according to their disease since it was reported to be higher in patients with acute respiratory failure than in neurologic patients^4^.

In this study, SOFA and days of endotracheal intubation were risk factors for mortality and even the regression tree analysis identified SOFA score at the day of tracheostomy and age as predictive risk factors for mortality in ICU. Previous studies found that SAPS II and APACHE III were risk factors for mortality in patients tracheostomized in ICU^13–15^. According to our regression three the clinical severity of patients may be a determinant factor for mortality and may be considered by clinicians in the choice for tracheostomy and in the discussion with families. Other studies reported that severity of illness on admission and pre-morbid functional status are associated with worse short- term mortality, whereas age and comorbidities assessed with the Charlson comorbidity score reflect negatively against long-term survival^13^.

Patients with tracheostomy were less likely discharged at home but at hospital facilities or rehabilitative structures. Literature reported that almost 50% of patients with or after tracheostomy were discharged in long term care facilities while 20% of them were discharged at home. However, taking into account their complexity and potential vulnerability, patients with a tracheostomy were also at high risk for morbidity or unplanned readmissions^16,17^. In a study by Spataro et al. patients with tracheostomy had a 13% readmission rate due to tracheostomy-specific issue^17^. Interestingly in this study 35 patients were discharged with an armed cannula without the inner cannula. This may expose patients to different complications like the risk occlusion by mucus. According to this we suggest that patients who are discharged from ICU with tracheostomy should be carefully monitored and managed by healthcare professionals trained in the specific management of these patients.

Quality of life of patients with tracheostomy was severely compromised at 3–6 and 12 months when compared with patients without tracheostomy. The quality of life of tracheostomized patients after ICU discharge has been investigated in a limited number of studies including relatively few patients. According to Antonelli et al., tracheostomized patients had a moderate impairment of functional status^18^. Patients tracheostomized for acute respiratory failure had a poor functional status^19^. In the SETPOINT trial, stroke patients with tracheostomy had severely disability after 6 months^20^. Recently Vargas et al. reported that quality of life of tracheostomized patients was more compromised in patients with neurologic compared with respiratory disease^4^. Finally, patients with tracheostomy experienced more pain and discomfort when compared patients without tracheostomy. This is in line with the current data from the UK NKHS trust reporting that patients with tracheostomies have high levels of psychological distress and anxiety^21^.

## Limitations

The present study suffers of some limitations. Firstly, an inhomogeneous population of critically ill patients was included in the study but representing the real-life clinical management. Secondly, the study was performed in Italian ICUs, thus limiting the generalizability of our data to other countries. However, they provide information about outcome critically ill patients admitted and tracheostomized in ICU. Thirdly, we did not compare tracheostomized patients with non-tracheostomized patients in ICU. However, an analysis was performed comparing patients with or without tracheostomy after ICU discharge in the follow-up period. Fourthly, despite comparing patients with and without tracheostomy at each follow-up regarding quality of life, disability, and discharge, we lacked data on tracheostomy in non-survivors at each time point, making impossible the assessment of tracheostomy as risk factor for mortality after ICU discharge.

## Conclusions

In mechanically ventilated patients, elective tracheostomy is associated with high mortality increasing over time after ICU discharge, with poor quality of life, psychological distress, and difficult rehabilitation. SOFA score at the day of tracheostomy and age may have a decisional role for the choice to perform elective tracheostomy.

### Supplementary Information

Supplementary Information.

## Data Availability

The datasets generated and/or analyzed during the current study are not publicly available due to EC decision but are available from the corresponding author on reasonable request.

## References

[1] Vargas M, Servillo G, Arditi E, Brunetti I, Pecunia L, Salami D (2013). Tracheostomy in intensive care unit: A national survey in Italy. Minerva Anestesiol..

[2] Altman KW, Ha TN, Dorai VK, Mankidy BJ, Zhu H (2021). Tracheotomy timing and outcomes in the critically ill: Complexity and opportunities for progress. Laryngoscope..

[3] Vargas M, Pelosi P, Servillo G (2014). Percutaneous tracheostomy: It’s time for a shared approach!. Crit. Care..

[4] Vargas M, Sutherasan Y, Brunetti I, Micalizzi C, Insorsi A, Ball L (2018). Mortality and long-term quality of life after percutaneous tracheotomy in intensive care unit: A prospective observational study. Minerva Anestesiol..

[5] Young D, Harrison DA, Cuthbertson BH, Rowan K, TracMan Collaborators (2013). Effect of early vs late tracheostomy placement on survival in patients receiving mechanical ventilation. JAMA..

[6] Battaglini D, Premraj L, White N, Sutt AL, Robba C, Cho SM, Di Giacinto I, Bressan F, Sorbello M, Cuthbertson BH, Bassi GL, Suen J, Fraser JF, Pelosi P (2022). Tracheostomy outcomes in critically ill patients with COVID-19: A systematic review, meta-analysis, and meta-regression. Br. J. Anaesth..

[7] Marra A, Vargas M, Buonanno P, Iacovazzo C, Coviello A, Servillo G (2021). Early vs late tracheostomy in patients with traumatic brain injury: Systematic review and meta-analysis. J. Clin. Med..

[8] Battaglini D, Missale F, Schiavetti I, Filauro M, Iannuzzi F, Ascoli A (2021). Tracheostomy timing and outcome in severe COVID-19: The WeanTrach multicenter study. J. Clin. Med..

[9] Garrubba M, Turner T, Grieveson C (2009). Multidisciplinary care for tracheostomy patients: A systematic review. Crit. Care..

[10] Fernandez R, Tizon A-I, Gonzalez J, Monedero P, Garcia-Sanchez M, De-la-Torre M-V (2011). Intensive care unit discharge to the ward with a tracheostomy cannula as a risk factor for mortality: A prospective, multicenter propensity analysis*. Crit. Care Med..

[11] Mondrup F, Skjelsager K, Madsen KR (2012). Inadequate follow-up after tracheostomy and intensive care. Dan. Med. J..

[12] Cinotti R, Voicu S, Jaber S, Chousterman B, Paugam-Burtz C, Oueslati H (2019). Tracheostomy and long-term mortality in ICU patients undergoing prolonged mechanical ventilation. PLoS ONE.

[13] Kojicic M, Li G, Ahmed A, Thakur L, Trillo-Alvarez C, Cartin-Ceba R (2011). Long-term survival in patients with tracheostomy and prolonged mechanical ventilation in Olmsted county, Minnesota. Respir. Care..

[14] Pandian V, Gilstrap DL, Mirski MA, Haut ER, Haider AH, Efron DT (2012). Predictors of short-term mortality in patients undergoing percutaneous dilatational tracheostomy. J. Crit. Care.

[15] Mehta AB, Syeda SN, Bajpayee L, Cooke CR, Walkey AJ, Wiener RS (2015). Trends in tracheostomy for mechanically ventilated patients in the United States, 1993–2012. Am. J. Respir. Crit. Care Med..

[16] Vargas M, Pelosi P, Tessitore G, Aloj F, Brunetti I, Arditi E, Salami D, Kacmarek RM, Servillo G (2015). Percutaneous dilatational tracheostomy with a double-lumen endotracheal tube: A comparison of feasibility, gas exchange, and airway pressures. Chest..

[17] Spataro E, Durakovic N, Kallogjeri D, Nussenbaum B (2017). Complications and 30-day hospital readmission rates of patients undergoing tracheostomy: A prospective analysis. Laryngoscope..

[18] Antonneli M, Michetti V, Di Palma A, Conti G, Pennisi A, Arcangeli A (2005). Percutaneous translaryngeal versus surgical tracheostomy: a randomized trial with 1-yr double blind follow-up. Crit. Care Med..

[19] Engoren M, Engoren CA, Buderer NF (2004). Hospital and long-term outcome after tracheostomy for respiratory failure. Chest.

[20] Bösel J, Schiller P, Hook Y (2013). Stroke-related early tracheostomy versus prolonged orotracheal intubation in neurocritical care trial (SETPOINT): A randomized pilot trial. Stroke..

[21] Liney T, Dawson R, Seth J (2019). Anxiety levels amongst patients with tracheostomies. Br. J. Anaesth..

