# Supplementary material for: Follow-up short and long-term mortalities of tracheostomized critically ill patients in an Italian multi-center observational study
Source: Sci Rep. 2024 Jan 28;14:2319. doi: 10.1038/s41598-024-52785-y (PMC10822864; doi:10.1038/s41598-024-52785-y)

**Supplementary Material**

**Follow-up short and long-term mortalities of tracheostomized critically ill patients in an Italian multi-center observational study**

**Table of contents:**

Table S1: List of participating centers and local investigators [page 2]

Table S2: Procedural findings and types of tracheostomies [page 3]

Table S3: Intraprocedural early and late complications of tracheostomy [page 4]

Figure S1: Data about patients discharged from the ICU [page 5]

Table S4. Follow-up of the overall population, and in patients with and without tracheostomy at follow-up [page 6]

Table S5. Characteristics of patients with tracheostomy at 3- 6- and 12- months follow-up [page 7]

Figure S2. Frequency of responses at 3- 6- and 12-months follow-up of each dimension of the EQ-5D-5L questionnaire in patients with and without tracheostomy [page 8]

**Table S1:** List of participating centers and local investigators

| Ospedale Policlinico San Martino, IRCCS per l’Oncologia e le Neuroscienze, Università degli Studi di Genova; Genova; Prof. P. Pelosi - Dott.ssa I. Brunetti |
| --- |
| Università degli Studi di Napoli “Federico II”, Napoli; Prof. G. Servillo - Prof. M. Vargas |
| Università Cattolica del Sacro Cuore, Roma; Prof. M. Antonelli |
| Università G. D’Annunzio - ASL2, Abruzzo; Prof. F. Petrini |
| Università di Torino, Torino Prof M. Ranieri - Prof. P.P. Terragni |
| Università di Ferrara, Ferrara; Prof. C. A. Volta - Prof. S. Spadaro |
| Azienda Ospedaliera Universitaria, Careggi, Firenze; Dott.ssa M. C. Ferraro |
| Ospedale S. Salvatore - Università dell’Aquila Prof. F. Marinangeli - Dr. A. Ciccozzi |
| Istituto Nazionali Tumori, Milano; Dott.ssa L. Persiani |
| Ospedale Santa Corona, Pietra Ligure; Dott.ssa J. Caprio |
| Università degli Studi di Sassari, Sassari; Prof. L. Brazzi |
| Seconda Università degli Studi di Napoli, Napoli - Prof. C. Belluomo |
| Azienda Ospedaliera Sant’Anna, Como; Dott. S. Zerbi |
| Azienda Ospedaliera Arcispedale S. Maria Nuova, Reggio Emilia - Dott. G. Salati |
| Ospedale San Donato, Arezzo; Dott.ssa M.C. Olivieri |
| Presidio Ospedaliero Ravenna, Ravenna; Dott. M Fusari, Dott. G. Zani |
| Università degli Studi dell'Insubria - Azienda Ospedaliera Fondazione Macchi, Varese; Prof. P. Severgnini |

**Table S2:** Procedural findings and types of tracheostomies (n-%)

|  | **Survivors**  **(497 pts -71.6 %)** | **Non- survivors (110 pts - 15.8 %)** | **p-value** |
| --- | --- | --- | --- |
| Cricoid- I° tracheal ring | 20 (4.2%) | 4 (3.6%) | 0.782 |
| I°- II° tracheal rings | 235 (49.5%) | 59 (53.6%) | 0.456 |
| II°-III° tracheal rings | 174 (36.6%) | 44 (40%) | 0.531 |
| III°-IV° tracheal rings | 36 (7.5%) | 3 (2.7%) | 0.065 |
| Other tracheal space | 3 (0.6%) | 0 (0%) | 0.403 |
| Ciaglia multiple steps | 12 (2.5%) | 5 (4.5%) | 0.260 |
| Ciaglia single step | 212 (44.6%) | 31 (28.1%) | 0.001 |
| Ciaglia pneumatic dilator | 28 (5.9%) | 10 (9.1%) | 0.226 |
| Griggs technique | 30 (6.3%) | 12 (10.9%) | 0.094 |
| Rotational technique | 135 (28.4%) | 37 (33.6%) | 0.936 |
| Trans-laryngeal technique | 18 (3.8%) | 4 (3.6%) | 0.224 |
| Surgical tracheostomy | 35 (7.3%) | 12 (10.9%) | 0.038 |
| Others | 1 (0.3%) | 1 (0.3%) | 0.260 |

**Table S3:** Intraprocedural early and late complications of tracheostomy (n-%)

| **Intra procedural complications** | **Survivors**  **(497 pts - 71.6 %)** | **Non-survivors**  **(110 pts - 15.8%)** | **p-value** |
| --- | --- | --- | --- |
| Accidental extubation | 8 (1.6%) | 5 (4.5%) | 0.067 |
| Lost airway | 6 (1.2%) | 4 (3.6%) | 0.081 |
| Repeated tracheal puncture | 46 (9.2%) | 12 (10.9%) | 0.704 |
| Change to another technique | 5 (1%) | 1 (0.9%) | 0.893 |
| Difficult cannula placement | 37 (7.4%) | 6 (5.5%) | 0.396 |
| Seldinger false passage | 4 (0.8%) | 0 | 0.334 |
| Cannula false passage | 9 (1.8%) | 0 | 0.146 |
| Tracheal ring fracture | 32 (6.4%) | 11 (10%) | 0.238 |
| Minor bleeding | 76 (15.2%) | 20 (18.2%) | 0.585 |
| Major bleeding | 4 (0.8%) | 1 (0.9%) | 0.945 |
| Desaturation | 20 (4%) | 15 (13.6%) | 0.000 |
| Damage posterior tracheal wall | 3 (0.6%) | 2 (1.8%) | 0.219 |
| **Early complications** |  |  |  |
| Multiple tracheal ring fracture | 6 (1.2%) | 0 | 0.238 |
| Cannula displacement | 8 (1.6%) | 1 (0.9%) | 0.559 |
| Minor bleeding | 46 (9.2%) | 13 (11.8%) | 0.489 |
| Major bleeding | 4 (0.8%) | 2 (1.8%) | 0.355 |
| Desaturation | 9 (1.8%) | 7 (6.3%) | 0.009 |
| Subcutaneous emphysema | 1 (0.2%) | 4 (3.6%) | 0.000 |
| Pneumothorax | 0 | 0 | - |
| Pneumomediastinum | 2 (0.4%) | 1 (0.9%) | 0.515 |
| **Late complications** |  |  |  |
| Minor bleeding | 25 (5%) | 11 (10%) | 0.507 |
| Major bleeding | 8 (1.6%) | 4 (3.6%) | 0.184 |
| Desaturation | 11 (2.2%) | 11 (10%) | 0.000 |
| Stoma infection/inflammation | 7 (1.4%) | 3 (2.7%) | 0.350 |
| Tracheoesophageal fistula | 0 | 0 | - |
| Tracheo-innominate fistula | 0 | 1 (0.9%) | 0.036 |

**Figure S1:** Data about patients discharged from the ICU.

**Table S4.** Follow-up of the overall population, and in patients with and without tracheostomy at 3 - 6 - 12 months after ICU discharge.

|  | **Overall** | **Tracheostomy** | **No Tracheostomy** | **p-value** |
| --- | --- | --- | --- | --- |
| **3 months follow-up** |  | **169 pts – 46.2%** | **197 pts – 53.8%** |  |
| Gender  male, n (%)  female, n (%) | 232 (64.4)  128 (35.6) | 100 (59.2)  69 (40.8) | 138 (70.1)  59 (29.9) | 0.0296 |
| Age, years, median (IQR) | 64 (50-74) | 66 (53-75) | 62 (49-73) | 0.0190 |
|  |  |  |  |  |
| EQ-5D-5L VAS, median (IQR) | 40 (20-60) | 20 (10-43.75) | 50 (40-70) | <0.0001 |
| Motor activity, median (IQR) | 5 (3-5) | 5 (5-5) | 3 (2-5) | <0.0001 |
| Self-care, median (IQR) | 5 (3-5) | 5 (5-5) | 5 (1-5) | <0.0001 |
| Usual activity, median (IQR) | 5 (3-5) | 5 (5-5) | 5 (2-5) | <0.0001 |
| Pain/discomfort, median (IQR) | 3 (1-3) | 3 (2-4) | 2 (1-3) | <0.0001 |
| Anxiety/depression, median (IQR) | 3 (1-4) | 3 (2-4) | 2 (1-3) | <0.0001 |
| **6 months follow-up** |  | **63 pts -22.5%** | **217 pts – 77.5%** |  |
| Gender  male, n (%)  female, n (%) | 182 (63.9)  103 (36.1) | 27 (42.9)  36 (57.1) | 150 (69.1)  67 (30.9) | <0.0001 |
| Age, years, median (IQR) | 63 (49-74) | 70 (52-78) | 62 (48-73) | 0.0075 |
|  |  |  |  |  |
| EQ-5D-5L VAS, median (IQR) | 60 (30-80) | 30 (10-50) | 70 (50-80) | <0.0001 |
| Motor activity, median (IQR) | 3 (1-5) | 5 (5-5) | 2 (1-4) | <0.0001 |
| Self-care, median (IQR) | 3 (1-5) | 5 (5-5) | 2 (1-5) | <0.0001 |
| Usual activity, median (IQR) | 4 (1-5) | 5 (5-5) | 2 (1-5) | <0.0001 |
| Pain/discomfort, median (IQR) | 2 (1-3) | 3 (1.25-4.75) | 2 (1-2) | <0.0001 |
| Anxiety/depression, median (IQR) | 2 (1-3) | 3 (1-5) | 2 (1-3) | <0.0001 |
| **12 months follow-up** |  | **36 pts – 17.5%** | **170 pts – 82.5%** |  |
| Gender  male, n (%)  female, n (%) | 136 (65.7)  71 (34.3) | 17 (47.2)  19 (52.8) | 116 (69.5)  51 (30.5) | 0.0109 |
| Age, years, median (IQR) | 61 (44-71) | 63 (51-76) | 59 (42-70) | 0.0498 |
|  |  |  |  |  |
| EQ-5D-5L VAS, median (IQR) | 70 (40-85) | 25 (0-50) | 75 (50-90) | <0.0001 |
| Motor activity, median (IQR) | 3 (1-5) | 5 (4.25-5) | 2 (1-4) | <0.0001 |
| Self-care, median (IQR) | 2 (1-5) | 5 (5-5) | 1 (1-4) | <0.0001 |
| Usual activity, median (IQR) | 2 (1-5) | 5 (5-5) | 2 (1-4) | <0.0001 |
| Pain/discomfort, median (IQR) | 2 (1-3) | 3 (1-5) | 1 (1-2) | 0.0001 |
| Anxiety/depression, median (IQR) | 2 (1-3) | 3 (1-5) | 2 (1-3) | 0.0003 |

IQR=interquartile range.

**Table S5.** Characteristics of patients with tracheostomy at 3- 6- and 12- months follow-up.

|  | **3 months follow-up**  **Alive, n=367** | **6 months follow-up**  **Alive, n=285** | **12 months follow-up**  **Alive, n=207** |
| --- | --- | --- | --- |
| **Check of tracheostomy site, n (%)** | 165 (100) | 70 (100) | 33 (100) |
| >once/day, n (%)  once/day, n (%)  once/week, n (%) | 45 (27.3)  63 (38.2)  37 (22.4) | 10 (14.3)  23 (32.9)  14 (20) | 0 (0)  12 (36.4)  11 (33.3) |
| >once/week, n (%) | 20 (12.1) | 17 (24.3) | 10 (30.3) |
| **People who control the site** |  |  |  |
| Caregiver, n (%)  Nurse, n (%)  Medical doctor, n (%)  Otolaryngologist  Speech therapist  Speech language pathologist  Neurologist  Pneumologist  Anesthetist/Intensive Care doctor | 29 (19.3)  129 (86)  63 (19.3)  15 (10.5)  8 (4.7)  7 (4.7)  12 (8.1)  37 (24.8)  34 (23.1) | 26 (42.6)  46 (75.4)  15 (24.6)  10 (16.4)  1 (1.6)  1 (1.6)  3 (4.9)  17 (27.9)  5 (8.2) | 19 (52.8)  22 (61.1)  11 (30.6)  5 (13.9)  0 (0)  2 (5.6)  2 (5.6)  6 (16.7)  3 (8.3) |
| Others, n (%) | 6 (4) | 1 (1.6) | 2 (5.6) |
| **Type of tracheostomy cannula, n (%)** | | | |
| Armed  Not armed  Cuffed without inner cannula  Cuffed with inner cannula  Fenestrated  Not fenestrated | 3 (2.5)  118 (97.5)  14 (8.6)  148 (91.4)  12 (7.4)  150 (92.6) | 2 (3.3)  58 (96.7)  56 (93.3)  4 (6.7)  4 (6.7)  56 (93.3) | 1 (2.9)  34 (97.1)  4 (11.4)  31 (88.6)  6 (17.1)  29 (82.9) |
|  |  |  |  |
| **Accidental decannulation, n (%)** | 2 (1.2) | 1 (1.6) | 2 (5.6) |

**Figure S2**. Frequency of responses at 3- 6- and 12-months follow-up of each dimension of the EQ-5D-5L questionnaire in patients with and without tracheostomy. Each dimension rate ranges from 1=I have no problems to 5=I have extreme problems. FU=Follow-up. Tracheo: tracheostomy, No Tracheo: no tracheostomy


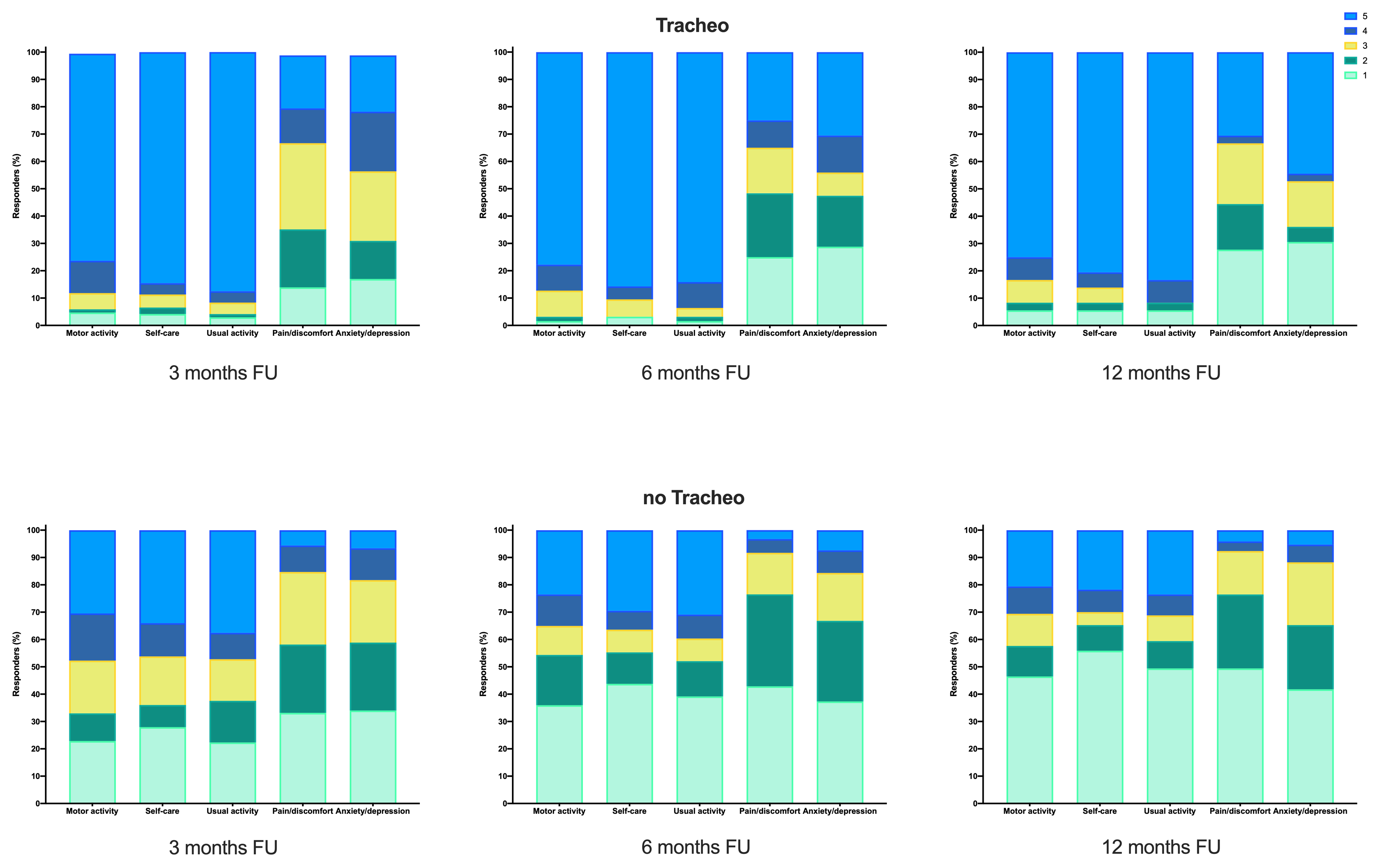

Supplement: Supplementary file 1 — Supplementary Information. [file 41598_2024_52785_MOESM1_ESM.docx]
